# Mitotic entry: Bora takes Polo to Aurora, and gives them a hug

**DOI:** 10.1038/s44318-025-00680-1

**Published:** 2026-01-28

**Authors:** Monica Gobran, Peter Lenart

**Affiliations:** Research Group Cytoskeletal Dynamics in Oocytes, Max Planck Institute for Multidisciplinary Sciences, 11 Am Fassberg, 37077 Göttingen, Germany

**Keywords:** Cell Cycle, Post-translational Modifications & Proteolysis, Structural Biology

## Abstract

Three new studies reveal structural details of the intricate interactions between Aurora A and PLK1 kinases and the co-factor Bora, and how they act together to trigger timely entry to mitosis.

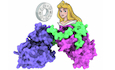

## Teaser

Cell division involves the complete reorganization of the cellular architecture, requiring precise coordination of cellular processes in space and time, but how does this control system work at the atomic level? Three new studies offer a glimpse under the hood, revealing structural details of the intricate interactions between Aurora A and PLK1 kinases and the co-factor Bora, and how they act together to trigger timely entry to mitosis.

Mitotic entry is a critical step in a cell’s lifetime; its precise execution is essential for maintaining genomic stability and preventing cancer. It is a bit like an airplane taking off: in preparation, checks are performed on internal and external systems, such as DNA damage or growth factor signaling. When ‘ready for departure’, the cell triggers a series of irreversible events in rapid succession: chromosomes condense, the nuclear envelope breaks down, the cell rounds up, and the microtubule cytoskeleton reorganizes to form the spindle. As in the case of take-off, at this stage swift and precise execution in the exact order and timing is critical.

At the theoretical level, seminal work by John Tyson and Béla Novák has outlined how the underlying control systems, the ‘sniffers, buzzers, toggles and blinkers’, may look like in the cell (Tyson et al, 2003). At the center lies a network of mitotic kinases, CDK1, PLK1, and Aurora A and B, which receive input from various intra- and extracellular signaling pathways and process and integrate these using intricate feedback mechanisms. The mitotic kinases then phosphorylate a plethora of substrates, triggering downstream cellular events.

A massive body of work over the past decades has mapped many molecular interactions in this regulatory network, also confirming the existence of multiple feedback mechanisms (Crncec and Hochegger, 2019). These results are often presented in the form of linear pathways or wiring diagrams visualizing the connectivity within the network—resulting in the somewhat false impression that we already understand it all. However, it is important to realize that such representations still lack critical details needed to *really* understand, quantitatively model, and accurately predict the behavior of such a control system. Firstly, we need to know the spatial and temporal dynamics, i.e., when and where in the cell these interactions take place. Secondly, we must understand these molecular interactions in their full biochemical and structural complexity. Three new papers published in *The EMBO Journal* and *EMBO Reports* by the Pintard, Musacchio and Bayliss laboratories (Pillan et al, 2026; Esposito-Verza et al, 2026; Miles et al, 2026) provide a beautiful example for the latter, revealing the intricate structural details of a key module in this regulatory circuitry.

The new studies focus on a branch of mitotic signaling that is essential to ensure timely entry into mitosis: here, the G2 cyclin-dependent kinase, CDK1-Cylin A, activates Aurora A, which in turn phosphorylates and activates PLK1. PLK1 then phosphorylates the phosphatase CDC25C, whose subsequent inactivation in turn activates mitotic CDK1-Cyclin B to promote timely entry into mitosis (Vigneron et al, 2018).

Mitotic kinases need to be activated by phosphorylation in the activation segment (T-loop) located in the hinge between their N- and C-terminal lobes, an event that leads to a conformational change stabilizing the kinase in the active form. This activating phosphorylation can be either via another kinase or through auto-phosphorylation (Bayliss et al, 2012). Aurora A can auto-phosphorylate Thr288 in its T-loop, but rapid dephosphorylation by counteracting phosphatases keeps Aurora A inactive during G2-phase (Zorba et al, 2014). Here comes the first unexpected twist: breaking this negative feedback requires a co-factor, the intrinsically disordered protein Bora (Hutterer et al, 2006; Seki et al, 2008). As shown by subsequent work, the upstream kinase CDK1-Cyclin A does not directly phosphorylate Aurora A, but rather modifies Bora (Vigneron et al, 2018). Phosphorylated Bora then forms a complex with Aurora A, ‘lending’ one of its phosphosites, p-Ser112, to mimic Aurora A’s T-loop phosphorylation in trans, so that Aurora A becomes activated without actually being phosphorylated on Thr288 (Tavernier et al, 2021).

**Figure 1:**
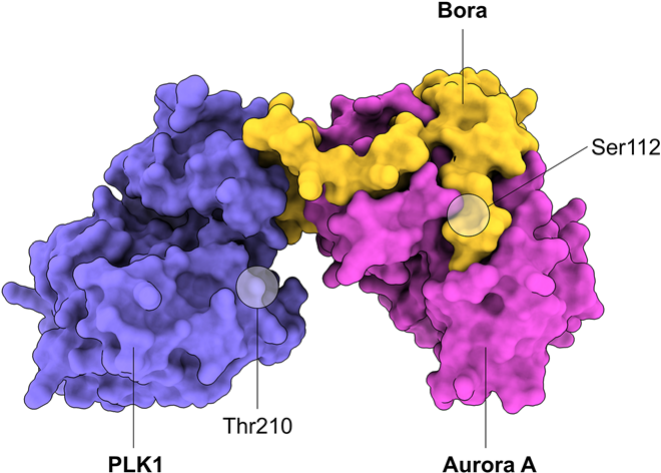
AlphaFold 3 model of the Bora-PLK1-Aurora A complex. Human Bora (residues 18–115, yellow), PLK1 (residues 36–330, purple) and Aurora-A (residues 128–392, pink).

For all three teams of authors, AlphaFold 3-aided modeling of the Aurora A-Bora interaction resulted in highly similar structures, revealing a number of intriguing new details of this interaction (Esposito-Verza et al, 2026; Pillan et al, 2026; Miles et al, 2026) (Fig. 1). Firstly, two motifs of Bora (M1 and M2) exhibit sequence similarity to the respective region of another Aurora A co-factor, the spindle assembly factor TPX2. This is followed by the Bora phosphodomain (M3) containing the Ser112 site. Indeed, Pillan et al (2026) show that a chimera, in which M1 and M2 are replaced by the TPX2 sequence (pTpx2-Bora), is capable of potentiating the activity of unphosphorylated Aurora A. This indicates that TPX2 and Bora share their mechanism of Aurora A activation, and confirms that Bora’s Ser112 can functionally replace Aurora A’s Thr288. Pillan et al (2026) further analyzed this interaction using an elegant bacterial reconstitution assay named MITOKINAC (MITOtic KINases Activation in *E. Coli*), showing that the interaction between Bora and Aurora A depends on hydrophobic residues in Bora’s M1 motif and Aurora A’s N-terminal lobe.

However, there is a second surprising turn: while both wild-type and chimeric Bora can fully activate Aurora A when bound to the pTpx2-Bora chimera, Aurora A is very ineffective in activating PLK1. PLK1 is normally activated by Aurora A-mediated phosphorylation on Thr210, so why is active Aurora A bound by the pTpx2-Bora chimera unable to carry out this function? Pillan et al (2026) show that Bora contains a unique conserved region (Bora 18-120) between the M1 and M2 motifs, which AlphaFold 3 models predict to form a ternary complex with the kinase domains of PLK1 and Aurora A. This complex lacks a direct interface between Aurora A and PLK1, as interactions are only seen between Bora and Aurora A and between Bora and PLK1. Thus, Bora literally bridges the two kinases, with the unique segment of Bora interacting directly with the αC helix of the PLK1 kinase domain (Fig. 1).

To validate the model including the probably transient ternary complex, which had not been experimentally observed before, Miles et al (2026) and Esposito-Verza et al (2026) introduced single amino-acid substitutions in Bora and PLK1, and assayed PLK1-Thr210 phosphorylation as a readout. Both groups consistently identified Phe56 and Trp58 as residues critical for the interaction between Bora and PLK1, and required for efficient Aurora A-dependent phosphorylation of PLK1 on Thr210. Esposito-Verza et al (2026) and Pillan et al (2026) further tested these conclusions in physiological models, using mammalian cell cultures and *Xenopus* egg extracts, respectively: confirming the in vitro results, mutants of Bora on Phe56 and Trp58 failed to rescue the reduced PLK1 phosphorylation during mitotis.

Why does the Aurora A-Bora pair phosphorylate Thr210 on PLK1 so efficiently? Esposito-Verza et al show that PLK1 contains a ‘gatekeeper’ residue, Lys208, in the PLK1 activation loop, which renders PLK1 a rather poor substrate for Aurora kinases, other than the Aurora A-Bora complex. Interestingly, this same residue appears to be responsible for protecting PLK1’s Thr210 from dephosphorylation by phosphatases after Bora is degraded later in mitosis (Chan et al, 2008; Seki et al, 2008). Miles et al identified another interesting site on Bora: they found that phosphorylation of Bora’s Ser59 by Aurora A enhances the interaction between Bora and PLK1, increasing the efficiency of phosphorylation of PLK1 by Aurora A.

What makes these studies so exciting? There are at least two main reasons. Firstly, these new works provide potential explanations for how spatial and temporal specificity in kinase activities may be achieved during mitotic entry. Aurora A activation is controlled by several co-factors: Bora and TPX2 as discussed above, but there are more, such as CEP192 or TACC3. Each of these co-factors localizes Aurora A to specific cellular structures, TPX2 to the spindle microtubules, CEP192 to the centrosomes, TACC3 to the spindle pole (Joukov, De Nicolo, 2018). Additionally, similar to TPX2, these co-factors are also expected to bind the same sites on Aurora A as does Bora. This implies that co-factors likely compete for Aurora A binding. Through these mechanisms, Aurora A activity can be efficiently restricted to specific cellular locations, while competition may ensure sequential action, i.e., temporal ordering of events during mitosis. These studies may also explain in structural detail how the same co-factors are involved in spatially and temporally restricting PLK1 activity, as reported recently (preprint: Dwivedi et al, 2025).

Secondly, from the methodological perspective, these studies demonstrate the enormous potential of AlphaFold structural modeling in cell-cycle research and generally for understanding kinase signaling pathways. Weak and transient interactions, multi-subunit complexes, and intrinsically disordered domains are very common features of these signaling networks, rendering them difficult to access by structural and biochemical methods. The above studies beautifully illustrate how structural modeling can serve as a rich source of testable hypotheses, as well as a platform to integrate experimental data validating these hypotheses—from NMR data to biochemical assays and mutational analyses. We strongly believe that these advances in structural modeling combined with other technologies, phosphoproteomics in particular, will open a new era of cell-cycle research. Ultimately, this shall enable us to construct a quantitative, predictive model of the biochemical control system that times and orders events of cell division. This has not only relevance for understanding one of the most fundamental transitions in the lifetime of any eukaryotic cell, but also for development of potential new therapeutic strategies for cancer treatment.
